# The Power of Field-Flow Fractionation in Characterization of Nanoparticles in Drug Delivery

**DOI:** 10.3390/molecules28104169

**Published:** 2023-05-18

**Authors:** Juan Bian, Nemal Gobalasingham, Anatolii Purchel, Jessica Lin

**Affiliations:** 1 Genentech Research and Early Development, Genentech Inc., 1 DNA Way, South San Francisco, CA 94080, USA; 2 Wyatt Technology Corporation, 6330 Hollister Ave, Santa Barbara, CA 93117, USA

**Keywords:** nanoparticle drug delivery systems, asymmetrical flow field-flow fractionation, light scattering detection, multi-attribute characterization

## Abstract

Asymmetric-flow field-flow fractionation (AF4) is a gentle, flexible, and powerful separation technique that is widely utilized for fractionating nanometer-sized analytes, which extend to many emerging nanocarriers for drug delivery, including lipid-, virus-, and polymer-based nanoparticles. To ascertain quality attributes and suitability of these nanostructures as drug delivery systems, including particle size distributions, shape, morphology, composition, and stability, it is imperative that comprehensive analytical tools be used to characterize the native properties of these nanoparticles. The capacity for AF4 to be readily coupled to multiple online detectors (MD-AF4) or non-destructively fractionated and analyzed offline make this technique broadly compatible with a multitude of characterization strategies, which can provide insight on size, mass, shape, dispersity, and many other critical quality attributes. This review will critically investigate MD-AF4 reports for characterizing nanoparticles in drug delivery, especially those reported in the last 10–15 years that characterize multiple attributes simultaneously downstream from fractionation.

## 1. Introduction

The application of nanotechnology for medical purposes has defined nanomedicine. Nowadays, nanomedicines such as nanoparticles (NPs) for drug and gene delivery have become an emerging field of medicine. Nanomedicines have significant potential to improve human health for prevention and treatment of diseases. Nanoparticles for drug delivery are revolutionizing the nanomedicine field, especially most recently, as several approved COVID-19 vaccines use nanoparticles to carry messenger RNA [1,2]. Additionally, nanoparticle-based vaccines and therapeutics in preclinical or clinical studies play an increasingly significant role against the COVID-19 pandemic [3]. The growing interest in applying NPs for drug delivery can be attributed to the appealing features such as improved stability and biocompatibility, enhanced permeability, and retention effect, as well as precise targeting [4,5,6].

Numerous NPs for drug delivery have been developed recently, including lipid-based NPs [7,8,9], polymer-based NPs [10,11,12], virus-like NPs [13,14], extracellular vesicles (EVs) [15,16], and inorganic NPs [17,18]. The chemical composition, stability, particle size distribution, nanoparticle shape, and morphology, as well as drug encapsulation and distribution, are critical parameters to characterize for nanoparticles [19]. In addition, regulatory agencies, including the U.S. Food and Drug Administration (FDA) and the European Medical Agency (EMA), have published multiple regulatory guidance documents to define the quality expectation for premarket submission [19,20,21]. Therefore, thorough understanding and characterization of nanoparticle drug delivery systems are critical for the identification of the critical quality attributes (CQAs) and successful development of nanomedicines.

To advance the development of the nanoparticles in drug delivery applications, gentle analytical techniques with full preservation of their native properties are in urgent demand. Field flow fractionation (FFF) is uniquely suited for the analysis of delicate nanoparticles because of the use of an external force field for gentle separation. FFF, which was invented and patented in 1966 by J. Calvin Giddings, applies an external field to an open channel to achieve separations [22]. The open channel design is highly conducive for separating fragile species with a wide particle size range, and offers the flexibility in carrier liquid selection [23]. Compared to commonly used size exclusion chromatography (SEC), the fundamental difference is the absence of a stationary phase in the FFF channel, which significantly reduces the system backpressure and shear force. This feature makes FFF noticeably gentler than SEC. The absence of a stationary phase as in liquid chromatography (LC) makes FFF a “soft” fractionation technique with minimal shear or mechanical stress towards analytes [24]. Another feature of FFF is the applicability for particles across a wide size range (1 nm to 10 µm) [23].

FFF is a group of distinctive techniques that use different types of separation fields perpendicular to the open channel. Thermal FFF (ThFFF) utilizes temperature difference across the channel to create the thermal gradient necessary to induce the separation [25,26]. Sedimentation FFF (SdFFF) uses sedimentation induced by gravity or a centrifugal force to separate particles [27,28]. Magnetic FFF (MgFFF) separates analytes according to their difference in magnetic properties [29]. Electrical FFF (ElFFF) introduces a transverse electrical current to create an electric field [30]. Electrical asymmetrical flow (EAF4) was developed afterwards as a variation of ElFFF to combine the electric field with the crossflow field [31]. Flow FFF (FlFFF) applies a crossflow field to facilitate separation and is the most versatile sub-technique of FFF. FlFFF has various formats, namely symmetric flow FFF (SF4), asymmetric flow FFF (AF4), or hollow fiber flow FFF (HF5), which differ in the geometrical channel shape and the way the crossflow is applied [32,33,34]. Table 1 summarizes the main FFF sub-techniques, the corresponding separation field, and the critical physicochemical properties of analyzed samples as the basis of separation.

**Figure 1:**
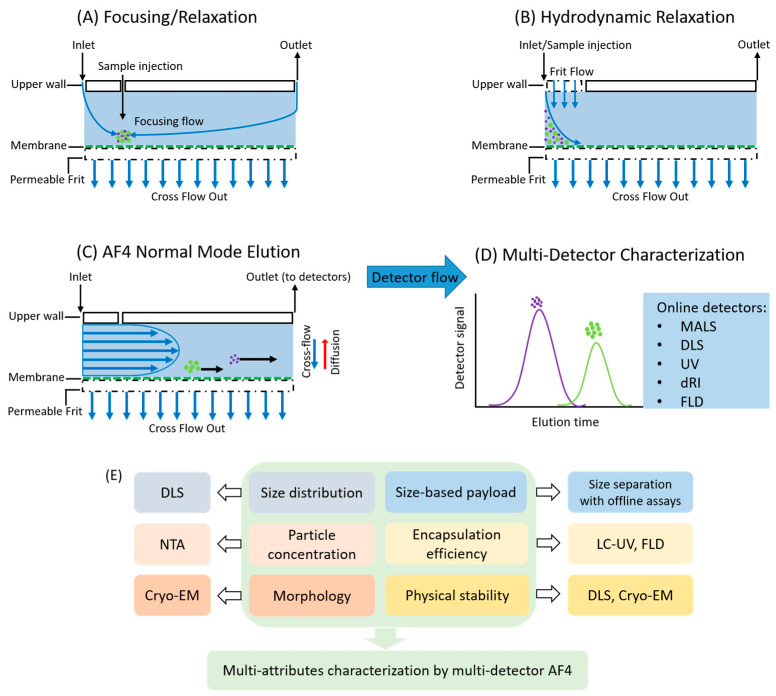
Representative diagram of relaxation and elution process in AF4: (**A**) focusing relaxation in conventional AF4; (**B**) hydrodynamic relaxation in frit inlet or dispersion AF4; (**C**) elution in the channel, showing the sample migration along with the parabolic flow, where smaller species travel faster than larger species; (**D**) following AF4 separation, the multi-detector system that is coupled with AF4 enables online characterization towards size-resolved fractions (Purple: population of particles with smaller size, green: population of particles with larger size); (**E**) critical quality attributes of the nanoparticle drug delivery systems and corresponding analytical techniques; multi-attribute characterization can also be achieved by the multi-detector AF4 (MD-AF4) system. MALS, multi-angle light scattering; DLS, dynamic light scattering; UV, ultraviolet; RI, refractive index; FLD, fluorescence detector; LC, liquid chromatography; NTA, nanoparticle tracking analysis; Cryo-EM, cryogenic electron microscopy.

Among these FFF sub-techniques, AF4 is the most commonly used because of its broad separation range, great versatility, and wide commercial availability, indicated by the dominance in scientific publications. The principle of AF4 has been reviewed previously [23,24,32,35,36]. In an AF4 setup, separation of analytes of different hydrodynamic sizes is achieved by an applied crossflow perpendicular to the separation channel, which is built from two blocks, one of which contains a semi-permeable membrane supported by a porous frit. The main separation zone is defined either with a spacer of defined thickness or can be built into the top block and influences the parabolic flow profile within the channel perpendicular to the crossflow. The semi-permeable membrane retains the analytes, while allowing the mobile phase to traverse. Then, the particles migrate along the parabolic laminar flow of liquid carrier and a dynamic equilibrium is established, where smaller particles (with a higher diffusion coefficient) equilibrate toward the middle of the AF4 channel (with higher velocity) and elute earlier than larger particles, as illustrated in Figure 1. Based on the sample relaxation prior to separation, two types of channels are currently available in the market for AF4 technology: focusing and hydrodynamic relaxation [37]. In the conventional AF4 channel, the sample is relaxed close to the membrane during the focusing step (Figure 1A), while the frit inlet channel or dispersion inlet channel uses hydrodynamic relaxation where no focusing is needed [38] (Figure 1B). The separation in AF4 can theoretically benefit from “focusing”, which reduces band broadening and improves resolution and efficiency. However, the focusing step might cause sample loss in some cases due to the adsorption on the membrane or aggregation of the sample, while sample loss on the membrane is typically negligible once the elution mode starts [39]. Therefore, frit-inlet FFF can be a preferred approach for particles that suffer from sample aggregation or undesirable membrane interaction. For example, the colloidal particle stability and particle size alterations were noted during the focusing step using a conventional AF4 channel, which could be circumvented by using a frit-inlet channel [40].

The critical quality attributes of nanoparticle drug delivery systems are usually determined through individual detection techniques (Figure 1E). As an elution technique, AF4 can be coupled to multiple detectors to enable multi-attribute characterization of the size-resolved fractions. Multi-angle light scattering (MALS) is capable of determining molar mass and root-mean-square radius or radius of gyration (R_g_). The range of R_g_ measured by MALS is ~10 nm to 500 nm, and even up to 1000 nm assuming shape-specific models and sufficient angular coverage [41]. In addition, particle concentration can be derived from the scattered intensity from MALS, and the lower limit for particle concentration is 10^7^ particles per milliliter for MALS analysis, below which it could result in noisy MALS peaks due to particle fluctuation [42]. Dynamic light scattering enables the measurement of Stokes radius or hydrodynamic radius (R_h_). R_h_ can be measured accurately using batch DLS from 0.2 to 1000–5000 nm depending on the instrument. However, the upper limit of online DLS coupling with FFF depends on the flow rate and detection angle, and assuming a flow rate of 0.5 mL/min and appropriate configuration, R_h_ can be determined accurately from 0.5 nm to ~300 nm [41]. With both online MALS and DLS, particle structure and morphology can be evaluated by the shape factor, which is the ratio of the R_g_ and R_h_ [43]. Ultraviolet (UV) and refractive index (RI) detectors, as well as fluorescence detectors (FLD), serve as concentration detectors depending on the analyte properties. The combination of dual-concentration detectors with MALS further expands the analysis. For example, UV and RI detector together with MALS enables the determination of molar mass for each component and the composition ratio of the complex nanoparticle drug delivery systems. This approach has been applied to analyze drug-loaded liposomes [44] and virus-like particles with nucleic acids [45]. Additionally, nanoparticle tracking analysis (NTA) and cryogenic electron microscopy (cryo-EM) are often used offline with AF4 for complementary particle quantitation and structure/morphology visualization.

Applications of FFF in environmental matrices [46], food macromolecules [47], pharmaceutics and biopharmaceutics [48], and nanomedicines [24,49] were reviewed previously. This article briefly revisits different FFF sub-techniques and the basic principles, and the following sections will focus on discussion of the recent applications of FFF, especially AF4 in the characterization of nanoparticles used in the drug delivery. Challenges and opportunities of FFF will also be outlined in this review.

## 2. Applications of FFF in Nanoparticle Drug Delivery Systems

One of the main challenges in developing nanoparticle drug delivery systems is particle characterization. Analytical scientists always need to consider the tradeoff between the level of details that the sample can be characterized with and the complexity of the analytical tools. For example, batch DLS offers a very quick and simple way to characterize particle size but lacks resolution and sensitivity to dispersity. On the other hand, cryo-EM provides an unprecedented level of detail but at a cost of much lower throughput and a limited sampling amount for statistical significance. Recently, FFF has gained increasing popularity for various nanoparticles owing to its separation capability towards a wide particle size range. FFF, especially AF4, has found plentiful use in the fields of nanomedicines and nanomaterials [32,49]. Coupling AF4 with flow-detectors, such as light scattering detectors (MALS, DLS) and concentration detectors (UV, RI, FLD), allows both in-depth understanding of structural information and quantitative measurement of the size-based distributions. To reveal the complexity, nanoparticles are separated by AF4 followed by online or offline detectors. For example, nanoparticle samples can be separated using AF4 and sent into a set of flow-through detectors for simultaneous online characterization or collected into fractions for offline characterization. Coupling with online detections has apparent advantages, as it does not cause agglomeration or breakage, nor alter NPs structure during the characterization. Among the online flow detectors used in combination with AF4, MALS and DLS are the most popular choices because they are size-based techniques that couple easily with a size-based separation [50]. In addition, spectroscopy detection (UV/FLD) is informative beyond quantitation, revealing information such as size-dependent plasmon shift and the location of labelled species [51,52]. Lastly, a combination of online and offline approaches can be used in parallel to provide orthogonal measurement.

This section is intended to provide a detailed overview of the recent applications using FFF, especially AF4, in the most common nanoparticle drug delivery systems. Our goal is to provide insights in the nanoparticle characterization when the FFF technique is combined with various detectors. Most importantly, the application sections highlight the use of multi-detector AF4 (MD-AF4) towards the multiple attribute characterization, including size distribution, shape or morphology, stability, drug encapsulation, and drug release. While the emphasis is mostly on AF4, which is the most widely used FFF technique, it is noteworthy to mention alternative FFF techniques, such as frit-inlet AF4 or HF5, for the characterization of different nanoparticles. Table 2 summarizes the FFF applications in different types of drug delivery nanoparticles discussed in this review, including the FFF technique, additional characterization techniques, and critical results.

### 2.1. Lipid-Based Nanoparticles

Liposomes are small artificial vesicles of spherical shape that are comprised of one or more concentric bilayers encapsulating an aqueous core. Due to their amphiphilicity, biocompatibility, and appropriate particle size, liposomes have been widely used in the past 40 years as membrane modeling, drug delivery vehicles, and nanoreactor vessels [76,77,78]. The first liposomal drug formulation ever approved by the FDA in 1995 was PEGylated liposome-encapsulated doxorubicin (Doxil^®^) to treat Kaposi’s sarcoma [79]. Nowadays, a large variety of liposomal-formulated drugs are approved by the FDA for clinical use [80]. Critical quality attributes of liposomal drug products include particle size distribution, charge, and payload amount. For instance, it has been shown that while smaller liposomes can decrease recognition by the complement system and innate immunity, thus enhancing the bioavailability, larger liposomes can increase drug payload [81,82].

**Figure 2:**
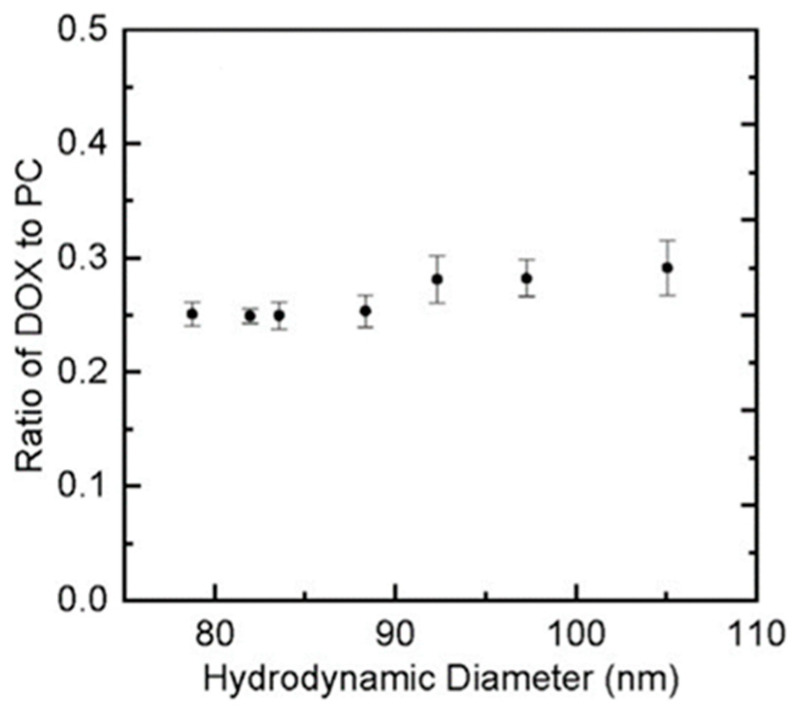
The mass ratio of DOX and total lipids as a function of the number averaged hydrodynamic diameter of DLF-1. Reprinted from Ref. [53] with permission from Elsevier.

These critical quality attributes of liposomes can be characterized using FFF as a separation technique followed by either online or offline detectors like discussed earlier. For example, Ansar et al., used AF4 for size-based fractionation of doxorubicin liposomal formulations, followed by offline NTA for particle sizing of the collected fractions, and online LC-MS for lipid and payload quantification [53]. Interestingly, this study concluded that the formulated liposomes had a narrow size distribution without any significant variation in D10, D50, and D90 values, and the drug to lipid ratios remained constant as a function of particle size, indicating that the drug loading to the liposomal particles is size independent. These findings are summarized in Figure 2, where the amount of DOX drug stays constant relative to the nanoparticle size.

Lavicoli and coworkers used AF4 coupled with MALS/DLS to study the peptide-liposome interaction [54]. They were able to characterize, in a single analysis, the selectivity of the peptides, the amount of peptides bonded to each liposome, and the induced change in the size distribution and morphology of the liposomes. By adding MALS and DLS downstream of the separation, AF4 provided information on particle shape and morphology by measuring their shape factor, which allowed them to identify subtle differences in complexes between positively charged F-AmP peptides with both the negatively charged POs (palmitoyloleoylphosphatidylcholine) and DL-AUVs (dilaurylphosphatidylcholine-anionic unilamellar vesicles). Huclier-Markai et al., used AF4 with MALS and a gamma ray detector to monitor the liposome size together with the incorporation of the high energy alpha emitter (^212^Bi) [55]. Considering 212Bi’s short half-life, it can only be delivered using labelled carrier particles (most notably, liposomes) that would rapidly accumulate in the target tumor. Animal studies suggested that the in vivo biologic period for the alpha-emitter is around 14 h, during which the metal must stay encapsulated. The AF4-gamma ray analysis has proven that more than 85% of radionuclides were retained in liposomes after incubating for 24 h at 37 °C in human serum. These results confirm that liposomes with a diameter of 100 nm represent a good vesicle to transport radionuclides for applications in targeted alpha therapy.

AF4 conditions have been investigated to study their influence on liposome fractionation. For instance, Hupfeld et al., studied the effect of ionic strength and osmolality of the carrier fluid in AF4 [76]. It was discovered that the liposomes eluted at different times when the ionic strength in the carrier fluid was changed. This was explained by osmotic stress-induced changes in vesicle size. The osmotic stress-induced size change in the liposome was found to be size dependent. Larger liposomes appeared to shrink or swell when exposed to hyper- or hypo-osmotic media, respectively. Smaller liposomes tend to shrink but not to swell under the same conditions. This study confirms the necessity to adjust the ionic strength of the carrier fluid to reduce inter-liposomal repulsion and interaction between liposomes and the FFF channel accumulation wall. Additionally, the osmotic pressure of the carrier liquid should be adjusted to match the pressure inside of liposomes using non-ionic additives. Kuntsche et al., evaluated the effect of fractionation conditions (flow profiles, injection volume, buffer composition) on the liposome and payload recovery [83]. The importance of osmolality match between liposome inner solution and carrier fluid was also confirmed. However, hydrophobic drug recovery had a strong dependence on its octanol-water partition coefficient. Because the sample is highly diluted during the fractionation, an alteration in the sample composition has to be studied and taken into consideration.

These newly discovered effects of AF4 elution conditions on sample composition have been utilized in developing drug release assays in several research studies [56,57,58]. Hinna et al., demonstrated that AF4 could be used for the drug transfer assay to quantify the retention of lipophilic compounds within liposomal carriers in the presence of lipophilic biological sinks [56]. The approach was extended for stability assessment of liposomes against the intestinal bile salts in applications for oral drug delivery, and it was found that the addition of taurocholate to egg-PC liposomes led to the formation of mixed-micelles and leakage of the calcein drug encapsulated inside liposomes [57]. Additionally, Holzschuh et al., introduced a novel approach to measure liposome-plasma protein interactions based on size by employing AF4 coupling with online detectors that enabled a simultaneous analysis of the sample (e.g., size determination). The authors obtained a good separation profile for liposomes and three main acceptor domains (albumin, HDL, and LDL) [58]. This study confirmed that rigid liposomes and PEGylated fluid liposomes showed higher stability in human plasma when compared to non-PEGylated fluid liposomes. This means that the bilayer composition of a liposomal formulation plays a significant role in stability and drug release in biological media. Hence, separation of the plasma-liposome sample by AF4 seems to be a potential alternative to already established methods.

Lipid nanoparticles (LNPs) are typically spherical nanoparticles with a solid lipid core that act as a novel pharmaceutical drug delivery system [84,85]. LNPs were first approved as a drug delivery vehicle in 2018 for the drug Onpattro to treat polyneuropathy in patients with hereditary transthyretin-mediated amyloidosis [86]. It became more widely known in late 2020 because some COVID-19 vaccines, notably mRNA-1273 [87] and BNT162b [88], used PEGylated-lipid nanoparticles for mRNA delivery [89]. Similar to liposomal drug formulations, determination of payload content relative to LNP size can be important to understand the efficacy and safety. AF4 has been successfully used for the separation and characterization of lipid-based drug delivery systems; however, electrostatically interacting LNP complexes with the relatively labile lipid-monolayer coating are more prone to destabilization during the focusing step in the conventional AF4 channel [90].

**Figure 3:**
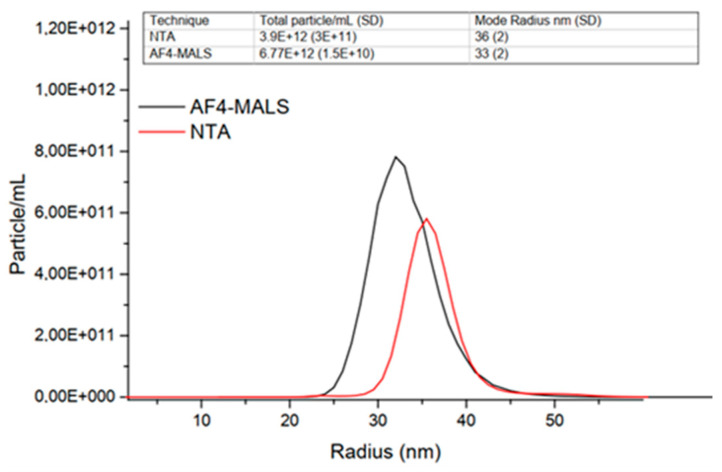
Number-based particle size distribution obtained by NTA and AF4-MALS. Total particle/mL concentration and radius are reported in the table in the graph. Reprinted from Ref. [39] with permission from Elsevier.

Non-focusing AF4 channels (frit-inlet or dispersion channel) can circumvent the instability of the LNPs during conventional AF4 separation. In a recent study, Mildner et al., demonstrated AF4 with the frit-inlet channel was well-suited for the analysis of lipid-based nanoparticles for RNA delivery with satisfactory reproducibility and sample recovery [39]. Downstream characterization by multi-detectors would benefit from the high sample recovery from AF4 separation, which makes AF4 become compliant with ISO/TS 21362:2018 (nanotechnologies-analysis of nano-objects, using asymmetrical-flow and centrifugal field-flow fractionation), resulting in better alignment with orthogonal techniques. As shown in Figure 3, particle concentration from MALS following the AF4 separation agrees well with batch NTA characterization. Nevertheless, AF4-MALS provides data across a wider radius distribution compared to batch characterization techniques.

**Figure 4:**
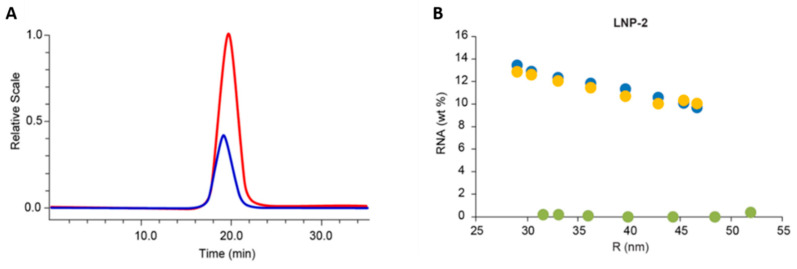
(**A**) UV chromatogram of empty LNP-2E (blue) and RNA-filled LNP-2F (red) showing the significant UV signal from LNP-2E due to the scattering phenomenon in the UV detector. (**B**) Size-dependent RNA distribution in LNPs. Average of duplicate fractionation and offline RPLC analyses (yellow) vs. data from online analysis (blue for RNA-LNP and green for empty LNP). Reprinted from Ref. [92] with permission from Elsevier.

It has been shown that the in vivo potency and tissue-penetration ability of LNPs are related to particle size [91]. Researchers from Merck & CO. enabled the online determination of the size-dependent RNA content in LNPs, which was validated through RNA quantitation using a reserved-phase liquid chromatography (RPLC) assay performed on individual size fractions [92]. This study involved an optimized MD-AF4 analysis with a patented UV scattering correction approach, which eliminates overestimation of UV absorption at 260 nm caused by the scattering of LNPs. Figure 4A demonstrates the significant contribution of UV scattering to the apparent absorption of 260 nm light for unloaded LNPs with no chromophore. Interestingly, after UV scattering was removed using the correction algorithm, the calculated RNA weight percentages for four different LNP formulations were found to be in excellent agreement with the data obtained by offline RPLC analysis of collected AF4 fractions. Figure 4B demonstrated the distribution of RNA content for one LNP formulation (LNP-2) using both online and offline approaches. The authors envisioned the potential for this application in QC environment to evaluate the total RNA content in LNPs within a specified size range, which is one of the critical quality attributes for RNA-LNP products.

### 2.2. Polymer-Based Nanoparticles

Polymer-based nanoparticles for drug delivery have many advantages because of their versatility, customizability, and broad variety of structure-function relationships [93]. These structures can include nanoparticle capsules, micelles, polymersomes, dendrimers, and many other polymer nanoparticle complexes, which can be tuned to achieve tailored functions, such as targeted delivery, improved solubility, or desired biodegradability. Delivery mechanisms can vary from direct conjugation of the drug to polymer (either covalently or ionically), physical adsorption to the carrier, or encapsulation [94]. The consequence of this versatility is that detailed characterization can be quite challenging because polymers are generally heterogeneous, which impacts their physiochemical properties, and their behavior in solution may vary from the solid state. Additionally, structure modification may inadvertently impact loading capacity, release rate, or efficacy [95]. This only further highlights the need for robust, reliable, and comprehensive characterization.

**Figure 5:**
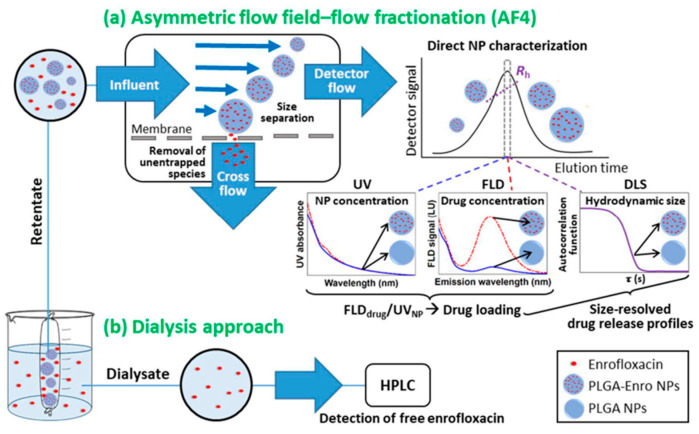
A scheme showing the convenience of AF4 for separating the unentrapped drug from the entrapped drug for determining drug loading. The free drug is removed via semi-permeable membrane via crossflow, while the entrapped drug elutes and is quantified. Reprinted from Ref. [59] with permission from Elsevier.

Polylactic-co-glycolic acid (PLGA) has been approved by the FDA for drug formulations and various therapeutic devices. PLGA NPs are biodegradable, biocompatible, and readily tunable by composition or by molar mass [96,97]. PLGA NPs can entrap drugs for drug delivery, and the particle size and shape can influence the drug loading. Shakiba et al., explored AF4 coupled with UV, FLD, and DLS to measure the release profiles for enrofloxacin entrapped in PLGA nanoparticles [59]. The AF4 methodology in comparison with the dialysis approach is provided in Figure 5. The combination of UV (for nanoparticle concentration), FLD (for drug concentration), and DLS (for size distributions) with AF4 provided comprehensive characterization, which was more streamlined and convenient than the traditional dialysis approach. Polysaccharides are another class of biodegradable polymers that are explored in drug delivery, which can load drugs by either covalent binding or entrapment [98,99,100]. The significant particle size distribution (25–150 nm radius or higher) and potential high molar mass (1–10 MDa) present analytical challenges during traditional SEC separation due to shear degradation [101,102]. Deng et al. explored AF4-MALS for the characterization of ultra-high molecular weight polysaccharides, highlighting the advantages of AF4 as a “soft” separation to ensure the integrity of the complex [60].

**Figure 6:**
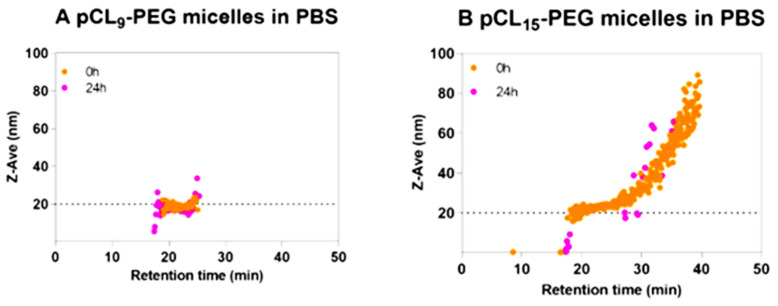
Hydrodynamic radius from in-line DLS after AF4 fractionation comparing micelle size distributions at different incubation periods. Reprinted from Ref. [61] with permission from Elsevier.

Polymer micelles are self-assembled colloids by amphiphilic polymers based on thermodynamic favorability at critical micellar concentrations, thus in aqueous media would present a hydrophobic core and an external hydrophilic shell. Challenges in the characterization of polymeric micelles are remarkable, as their stability is directly correlated to concentration, and the micelles may disassemble upon dilution (whether in the bloodstream or in analytical methods). Environmental factors like pH, temperature, and micelle composition can also affect their shape and morphology, leading to varying delivery efficiency [103,104]. Ideal fractionation methods should provide the distinct capability of separating intact micelles from disassembled micelles, unimers, and unencapsulated nanomedicine. However, traditional size separation methods like SEC have several limitations, such as micelles’ disassembly on the column, interacting with or adsorbing to the stationary phase, and likely not eluting out from the column [12]. In this case, AF4 is very well suited for micelle fractionation. For example, Liu et al., employed AF4 to investigate the in vitro stability of micelles in human plasma using both empty micelles and those loaded with tetra(hydroxyphenyl)chlorin (mTHPC) [61]. They explored both covalently crosslinked and non-crosslinked micelles based on amphiphilic block copolymers with poly(ε-caprolactone), poly(1,2-dithiolane-carbonate), and/or poly(ethylene glycol). Micelles were prepared with and without mTHPC, and release was studied by incubating loaded micelles and taking samples at various time points and running AF4 coupled to RI, FLD, and DLS referenced against empty micelles. Size distributions, achieved with inline DLS, helped to elucidate stability, and the results indicated covalently crosslinked micelles had much better stability than non-crosslinked micelles. A representative hydrodynamic radius distribution is provided in Figure 6.

Polymersomes are self-assembled hollow nanostructures that are analogous to liposomes and capable of forming spherical or non-spherical shapes [105]. Compared to micelles, polymersomes are in the form of a bilayer with a solvent core for drug encapsulation (i.e., aqueous core for water-based assemblies). For aqueous systems, amphiphilic block copolymers generally form micelles when the hydrophilic polymer fraction is greater than 50%, and form polymersomes when the hydrophilic polymer fraction is between 25 and 45%. Because of the many unique shapes and structures that polymersomes can take, including rod-like assemblies, spheroids, discocyte, and stomatocyte structures, characterizing their size and morphology can be quite challenging [105].

**Figure 7:**
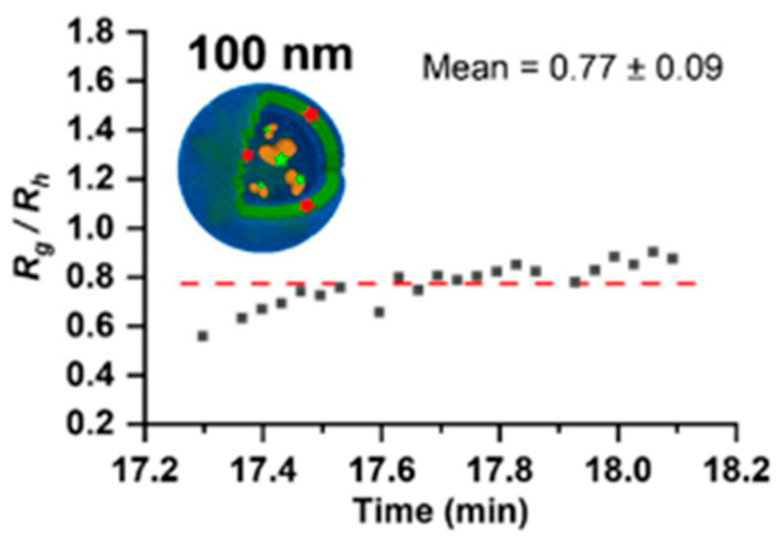
Ratio of R_g_ and R_h_ for spherical BSA-loaded polymersomes extruded using a 100 nm filter. The red line represents the mean value of these ratios. Reprinted from Ref. [62] with permission from American Chemical Society.

It has been demonstrated that the relationship between R_g_ and R_h_ can provide insight on the conformation of macromolecules. The ratio of R_g_/R_h_ defines the shape factor (ρ = R_g_/R_h_), which can be plotted as a Burchard-Stockmayer plot [106,107]. As a result, the ratio of R_g_/R_h_ approaches ρ = 1 for a theoretical hollow sphere with a thin shell, or ρ = 0.77 for a solid sphere. Wauters et al., explored polymersomes made of amphiphilic block copolymers based on polyethylene glycol and poly(D,L-lactide) (PEG-PDLLA), which were polymerized with various polymer chain lengths and block ratios to achieve spherical and cylindrical assemblies [62]. By analyzing the Burchard-Stockmayer plots from online MALS and DLS data, they were able to not only determine the size of the empty polymersomes, but also provide insights for the shape: whether the polymersomes were spherical or cylindrical. They also investigated polymersomes loaded with BSA or DiD (a far-red fluorescent small molecule) and used the shape factor derived from the Burchard-Stockmayer plot to evaluate if the polymersome was empty or filled. As plotted in Figure 7, BSA-loaded polymersomes showed a reduction of the R_g_ values, yielding an average ρ of 0.77 ± 0.09, indicating the presence of BSA inside polymersomes [62].

Dendrimers are branched polymers with a defined structure, typically hyperbranched polymers emanating from a central core [108]. Dendrimers are capable of forming scaffolds and cavities that lead to advanced complexes, allowing further functionalization to improve critical quality attributes like biocompatibility. Examples of dendrimers include polyamidoamine (PAMAM), polypropyleneimine (PPI), and several other amine- or ether-derivatives [109]. When characterizing dendrimers, it is critical to separate dendrimers from the impurities, including dendrimer defects with missing arms, entangled or aggregated dendrimers, and other suboptimal structures. Lee et al., explored the structural changes of PAMAM with AF4, including generational dispersity (inter-molecularly coupled dendrimers), skeletal dispersity (missing arms and molecular loops), and other structural defects that occur during synthesis [63]. Various analytical techniques have been tested for characterization of dendrimers, such as SEC, infrared spectroscopy, capillary electrophoresis (CE), nuclear magnetic resonance (NMR), and mass spectrometry, while their performance tends to be worse as the size of the dendrimer increases. However, AF4 was able to separate PAMAM dendrimers with optimized conditions (flow rate, pH, and salt concentration), including separation of four different dendrimer structures with a wide range of molecular weights of 14 to 467 kDa [63].

**Figure 8:**
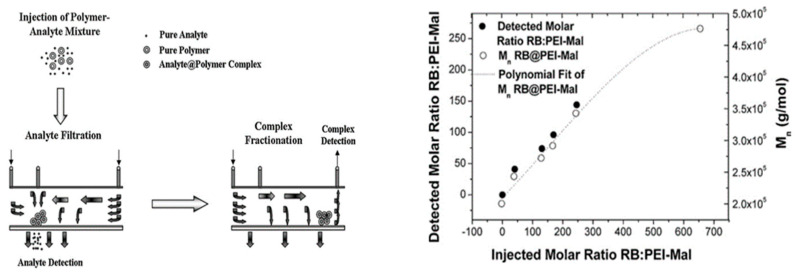
Conceptual diagram of executing free, small analyte detection via crossflow pathway detectors (**right**) and complex fractionation and subsequent detection (**left**). The molar ratio of RB:PEI-Mal in the complex was achieved by separation of free dye from complex. Reprinted from Ref. [64] with permission from Elsevier.

One of the more creative studies for evaluating the drug encapsulation comes from the work of Boye and coworkers, who installed a UV detector on the crossflow pathway of the AF4 to measure the free drug [64]. In this case, they studied hyperbranched PEI with a maltose shell (PEI-Mal) dendrimers complexed with a dye, Rose Bengal (RB). Traditionally, multiple detectors are installed downstream of the channel outlet, while the authors included a UV detector on the crossflow outlet. This innovative setup allows for measuring the concentration of small molecules that traverse the membrane and exit via what is normally the crossflow waste pathway, as illustrated in Figure 8. The authors investigated parameters like membrane material and molecular weight cutoff with pure RB and RB-PEI-Mal complex, and the free RB was quantified using a calibration curve established by the UV detector at the crossflow pathway. This work demonstrated the power of AF4 in fractionation and purification of a mixture of nanoparticles and small drug molecules, as well as free drug quantification by exploiting the semi-permeable membrane.

### 2.3. Viral Vectors and Virus-like Nanoparticles

**Figure 9:**
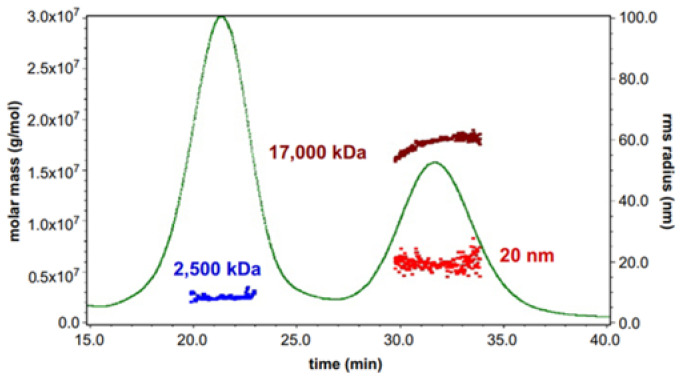
AF4 fractogram of the VLP with the radius and molar mass distributions of the two different size populations measured by MALS and RI signal. Reprinted from Ref. [45] with permission from Elsevier.

The biotechnology sector has been investing in viral vectors for gene therapy for many years now [110,111]. As of 2023, more than 3600 gene therapy clinical trial studies are ongoing or have been approved, and more than 70% of them are based on viral vectors [112,113]. Despite all this progress, commercial-scale production remains challenging, and the final viral particles that contain drug substances are not well characterized. As aforementioned, AF4 separation with downstream flow-through detectors (MALS, DLS, UV) is a very promising methodology for viral vector analysis. For example, Cirkowitz et al., have utilized AF4 to guide the development of the scalable process to produce virus-like particles (VLP) derived from the human polyoma JC virus, and then conducted scattering detector-based analytical characterization [45]. Figure 9 shows a fractogram that demonstrates the necessity of size-based separation because VLP expression in the insect cells produced not only desired VLPs (17,000 kDa), but also VP1 aggregates with a lower molecular mass (2500 kDa). Therefore, the use of AF4-MALS as an analytical tool enabled the development of a scalable process for the production, purification, and packaging of the VLPs based on the human polyoma JC virus.

Additionally, a combination of various modes of FFF can be used to better understand the complicated nanoparticle samples. A research group from the University of Utah in collaboration with Pfizer used AF4 in combination with ElFFF to obtain size and electrophoretic mobility of three bacteriophage-like VLPs: a blank Q beta bacteriophage, which is denoted as VLP, and two conjugated particles with different peptides [65]. The comparison of electrical and asymmetric flow modes of FFF revealed that separation of samples with similar size but different electrical properties can be achieved to a small extent. ElFFF showed consistent shoulder peaks in fractograms, indicating the presence of particle population with different surface charge properties. Additionally, this allows for the quantification of surface charge properties of polydisperse samples with multiple species present in the mixture.

### 2.4. Extracellular Vesicles

In addition to VLPs, cell-secreted nanoparticles, extracellular vesicles, also attracted increasing interest in the field of target drug delivery [114], and the remarkable advance in the development of EV-based drug delivery systems has been witnessed in the last decades [115,116,117]. EV is a cluster of heterogeneous lipid bilayer-delimited nanoparticles of different sizes, cargos, and surface markers, including exosomes, microvesicles, and apoptotic bodies. Exosomes are a subtype of EVs and are typically 30–100 nm in diameter, which is the smallest population in EVs compared to microvesicles (50–1000 nm in diameter) and apoptotic bodies (100 nm to several micrometers in diameter) [118]. EVs enable intercellular communication by serving as delivery vehicles for a wide range of endogenous cargo molecules, like proteins and nucleic acids. For instance, Zhang et al., transfected HEK293T cells with si-RNA (small interfering RNA) and incubated the isolated exosomes with gastric cancer cell lines. They demonstrated that exosome-delivered si-RNA could reverse chemoresistance to cisplatin in gastric cancer [119]. Additionally, exosomes have been used to incorporate small drug molecules with poor bioavailability to improve the delivery efficiency. In the study led by Pascucci et al., mesenchymal stromal cells (MSCs) were incubated with a high dosage of paclitaxel (PTX), a hydrophobic mitotic inhibitor with a powerful anticancer effect. Exosomes released by MSCs contained encapsulated PTX, showing stronger anti-proliferative activity than PTX alone towards pancreatic adenocarcinoma [120].

EVs as drug delivery systems present unique advantages, namely low immunogenicity and excellent biocompatibility and biostability. Currently, only two engineered exosome therapeutic candidates, both from Codiak BioSciences, have entered clinical development (ExoIL-12™ and ExoSTING™) [116]. To expand its industrial applications, the International Society for Extracellular Vesicles (ISEV) initiated the efforts “the minimal information for studies of extracellular vesicles” (MISEV) towards EV separation and characterization techniques in 2014, which was updated in 2018 and 2021, suggesting that the size distribution, morphology, purity, and stability of EVs should be investigated [16,121,122]. As the field continues to grow, a powerful separation technique coupled with online detectors is needed to obtain a full picture, where AF4-MALS could lend itself well to such applications. Despite very few applications of AF4-MALS directly towards EV drug delivery systems, researchers have already paved the way for the EV drug delivery characterization by using AF4-MALS for the characterization of various EV subtypes from different body fluids [66,123,124,125]. Thereby, AF4-MALS plays a promising role in separating and characterizing EVs in various settings, including drug delivery [126,127].

Sitar et al., used AF4-MALS-UV for size-based separation, characterization, and quantitation of exosomes by varying the AF4 parameters. They found the crossflow velocity and channel thickness significantly influenced the fractionation performance, whereas the focusing time had less impact [67]. AF4-MALS also showed broad size distribution and two subpopulations present in the exosome sample, larger exosomes and smaller vesicle-like particles. Batch NTA analyses were also conducted directly for the bulk exosome, and the results showed that NTA overestimates the size and the number density for the larger exosome population [67]. This issue has been reported previously for size measurement when applying light scattering towards heterogeneous suspensions. Large particles scatter more light than small particles, and if present in polydisperse samples, could potentially dominate the scattered light fluctuations and thus shift the particle size distribution and uplift the average diameter [128]. Therefore, AF4 became a powerful tool to address this issue by separating different extracellular vesicles prior to size characterization and quantitation. Oeyen et al., described a method using AF4-MALS-UV for characterization and quantitation of urinary EVs, where R_g_ defined by MALS was in the range of 40–160 nm. The online UV detector allows for the determination of contaminating proteins in the sample fraction. The study also demonstrated that AF4-MALS-UV was a highly reproducible technique compared to NTA, showing its potential as a reliable quality control method for EVs. It is noteworthy that authors proposed to include AF4-MALS-UV as a standard characterization method for EVs in the ISEV guidelines to improve the quality of the EV-related research [66].

**Figure 10:**
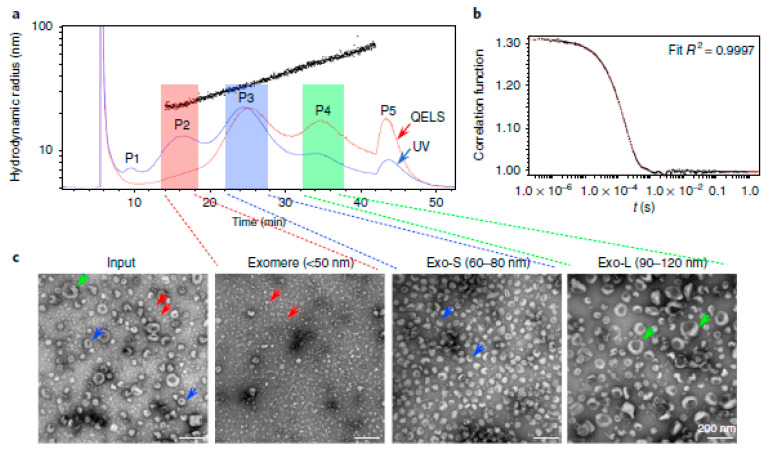
Separation and characterization of EVs using multi-detector asymmetrical flow field-flow fractionation (MD-AF4). (**a**) A representative AF4 fractionation profile of B16-F10-derived exosomes with UV and QELS (DLS) signals in blue and red separately; black dots illustrate hydrodynamic radius (Rh, nm), showing the particle size distribution over retention time. P1-P5 mark the peaks detected based on UV absorbance. Fractions were pooled for exomeres (hydrodynamic diameter < 50 nm), Exo-S (60–80 nm), and Exo-L (90–120 nm). (**b**) Representative correlation function in QELS for P3 (t = 25.1 min). (**c**) TEM imaging analysis of exosome input mixture (pre-fractionation) and fractionated exomere, Exo-S and Exo-L subpopulations. Arrows indicate exomeres (red), Exo-S (blue) and Exo-L (green). Reprint from Ref. [68] with permission from Springer Nature.

AF4 MALS/DLS-UV was also successfully used for the identification of small EV subpopulations and corresponding biophysical and molecular characterization [68]. The AF4 fractogram of B16-F10 melanoma-derived small EVs is displayed in Figure 10a. A total of two exosome subsets, including large exosome vesicles (hydrodynamic diameter 90–120 nm) and small exosome vesicles (hydrodynamic diameter 60–80 nm), as well as one abundant non-membranous nanoparticle termed “exomeres” (hydrodynamic diameter < 50 nm). Representative AF4 fractions were further analyzed by TEM, showing distinct morphology and size for each small EV subset (Figure 10c) [68]. Based on this study, Zhang et al., established a protocol for “asymmetric-flow field flow fractionation of small extracellular vesicles” consisting of four sections: I. Preparation of small extracellular vesicles (sEVs) from cell culture. II. AF4 fractionation of sEVs. III Online data collection and analysis. IV. Fraction collection, concentrations, and characterization [129]. This protocol provides general guidance for the EV separation using AF4, which makes AF4 more accessible and friendly to new users.

**Figure 11:**
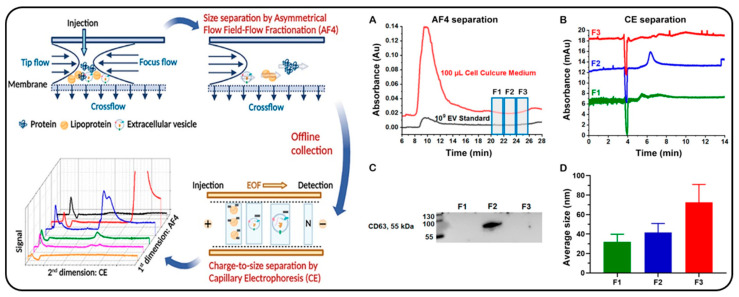
Representative workflow of offline coupling of AF4 and CE for separation of extracellular vesicles. (**A**) Fractograms of injection of HeLa cell medium (red trace) and standard EVs (black trace) to AF4. (**B**) CE traces of three AF4 fractions were collected from injection of 109 standard EVs, (F1: 20–22 min in green trace, F2: 22–24 min in blue trace, and F3: 24–26 min in red trace). (**C**) Western-Blot analysis of the CD63 protein, an EV marker in three AF4 fractions collected from injection of a HeLa cell medium. (**D**) Average diameter of the particles in the AF4 fractions collected from a HeLa cell medium observed in SEM. Reprint from Ref. [70] with permission from ACS publications.

Size separation of exosomes is critical for monitoring the size changes of EV subpopulations associated with various biological statuses. Moon’s group used AF4 for size sorting of exosomes, followed by exosome fraction collection and characterization by offline analytical tools [69,130]. For example, Joon-Seon et al., utilized AF4 to separate urinary exosomes by size, demonstrating a significant difference in exosome sizes between healthy controls and patients with prostate cancer [69]. Gao et al., highlighted the versatility of AF4 offline coupling with CE for EV analysis [70]. The authors demonstrated that EVs could be resolved from free proteins and high-density lipoproteins by AF4, which could be further separated from co-eluted low-density lipoproteins through CE by different surface charges (Figure 11). The AF4 fraction allowed for rapid EV quantitation in various samples in the matrix, showing the great potential of AF4 in reducing the matrix interference for the characterization of EV subpopulations produced by cell lines or present in clinical samples [70].

In addition to conventional AF4, efforts have also been made to separate and characterize EVs using alternative flow FFF techniques. Although HF5 plays a much less significant role compared to other flow FFF techniques due to the lack of flexibility and limited sample loading, its improved resolution, sensitivity, and disposability make it suitable for nanoparticles with limited sample volume and/or require disposable separation devices [23,131]. Marassi et al., separated different EV populations derived from the C2C12 cell line using HF5 followed by MALS-UV-FLD detection, which provided insights on the content of different EV subsets in addition to size distribution; for example, DNA/RNA was observed to release from the large EV populations while protein was detected from the small EV populations [71]. Derivative AF4 techniques were also evaluated in this field. EAF4 is another variant of AF4, which combines two complementary fields for separation. Drexel et al., described a method using EAF4-MALS combined with NTA through a flow splitter for the analysis of liposomes and exosomes in the biological matrix, where EAF4 provided online sample purification while simultaneously enabling access to size and Zeta potential and MALS and NTA detection added high resolution particle size and concentration information [72]. This study highlights the benefits of the EAF4-MALS-NTA platform to study the behavior of EV-based drug delivery vesicles under in vivo-like conditions.

### 2.5. Inorganic Nanoparticles

Inorganic NPs attracted increasing attention in the past decades because of their potential in carrying various therapeutic agents, such as small molecule drugs, peptides, proteins, and genes. When employed as nanocarriers, inorganic NPs have shown good drug loading capacity, stability, and biocompatibility [132,133]. The finely controlled size of the inorganic nanoparticles provides a versatile platform for drug encapsulation either in the cavity of the nanoparticle structure or on the surface of the nanomaterials due to the high surface-area-to-volume ratio [134,135]. The properties of the inorganic nanoparticles, including size, shape, and composition, could affect their performance in drug delivery [18,136]. The most investigated inorganic nanocarriers include gold nanoparticles (GNPs) and mesoporous silica nanoparticles (MSNs), and some of the inorganic nanocarriers are also investigated in the clinical trials [136,137,138,139,140]. Kong et al., reported the use of polyethyleneimine (PEI)-entrapped GNPs modified with peptide via a polyethylene glycol (PEG) spacer as a vector for B-cell lymphoma-2 (Bcl-2) siRNA delivery to glioblastoma cells [141]. Their results revealed that the modified GNPs could deliver Bcl-2 siRNA to the target cells with excellent transfection efficiency, leading to specific gene silencing in the target cells. In another study, the anticancer drug doxorubicin (DOX) was attached to GNPs with an average diameter of 30 nm through a pH-sensitive linker, which allowed for the intracellular triggered release of DOX from the GNPs once inside acidic organelles [142].

**Figure 12:**
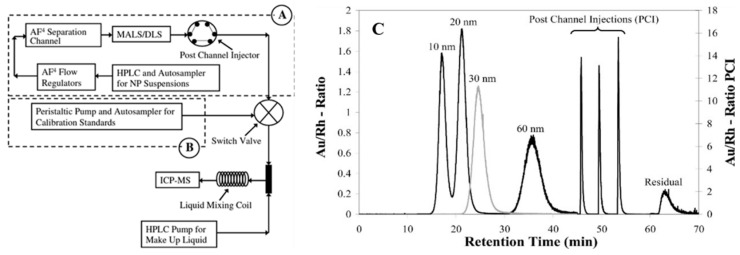
Representative diagram of AF4-MALS-DLS-ICP-MS platform including (**A**) the AF4-MALS-DLS system with post channel injection and (**B**) flow injection of calibrant solution. A switch valve allowed A or B to be operational and a separate HPLC pump delivered make-up liquid. (**C**) AF4-ICP MS fractogram of a mixture of 10, 20, and 60 nm Au NPs (black line) superimposed on a fractogram corresponding to 30 nm NIST Au NPs (light gray line). The signal intensities of post channel injections of 10, 20, and 60 nm Au NPs have been indicated on the secondary y-axis. Reprint from Ref. [73] with permission from ACS publications.

As the drug delivery system, inorganic NPs hold structural strength compared to organic NPs. Their surface is often coated by other materials to form hybridized framework, some of which can change their size or morphology to improve the drug loading [18]. Since the drug loading may alter due to framework disintegration, size, or morphology change, thereby, a size-indicating analytical method that allows structure and composition characterization is highly needed. In this context, AF4-MALS represents an exciting opportunity for inorganic nanoparticles for size separation and characterization. Schmidt et al., developed an analytical platform coupling AF4 with MALS, DLS, and inductively coupled plasma mass spectrometry (ICP-MS) to separate GNPs by size and quantitatively measure the GNP mass concentration (Figure 12) [73]. The authors successfully separated three GNP populations, which were quantified by ICP-MS with recovery within 50–95% [73]. In this study, to ensure the stability of GNPs during separation, SDS was added as a surfactant in the aqueous carrier to ensure the NP stability during the separation in AF4. The influence of the membrane was also tested to improve the GNP recovery and results demonstrated that the polyethersulfone (PES) membrane was superior to regenerated cellulose, resulting in higher recovery for the GNPs and better peak shape of GNPs in the fractogram [73]. Indeed, utilization of a representative medium as AF4 mobile-phase is critical for the separation and characterization of GNPs and their conjugates, as the properties of mobile phase (e.g., pH and ionic strength) can influence the electrostatic property of the nanoparticle samples and membrane in the channel [143,144]. Wang and coworkers found GNPs alone aggregated or precipitated in the AF4 channel when the ionic strength of the mobile phase was increased. However, when proteins were present, they formed a corona on the GNPs’ surface to increase the GNP stability, making ionic mobile phases such as phosphate buffer appropriate [143].

Lee et al., used the AF4-DLS to study the elution behavior of the GNPs with three different morphologies: gold nanospheres (GNS), gold nanotriangles (GNT), and gold nanorods (GNR) [74]. The authors found that although the diameter of the GNS was approximately similar to the length of the GNR from TEM, its elution time (3.7 min) was earlier than that of the GNS (4.5 min), which indicated that non-spherical particles move down the AF4 channel by different mechanisms compared to the spherical particles [74]. Additionally, nanosized metal-organic frameworks (nanoMOFs) were also investigated in drug delivery applications. Roda et al., used AF4-MALS-UV-RI system to study the MIL-100(Fe) nanoMOFs loaded with azidothymidine derivatives with three different degrees of phosphorylation: azidothymidine (AZT, native drug), azidothymidine monophosphate (AZT-MP), and azidothymidine triphosphate (AZT-TP) [75]. The gentle separation nature of AF4 allows for the detection of low abundance aggregation in the MOFs. The authors found that AZT-loaded nanoMOF had an identical PSD profile with the empty nanoMOF, confirmed by their similar scattering behavior, while AZT-MP and AZT-TP-loaded nanoMOFs showed increased scattering intensity and particle size compared to the empty ones [75]. Their findings through AF4-MALS also highlighted the key role of the phosphate group for improved encapsulation of AZT derivatives to nanoMOFs [145]. They successfully demonstrated the capability of AF4-MALS to provide evidence for particle size distribution and stability, as well as surface modification of the drug-loaded nanoMOFs.

## 3. Current Challenges and Future Trends

FFF coupled with light scattering detectors (MALS and DLS) as well as concentration detectors (UV, RI, FLD) has become the present-day analytical technique to tackle the unique challenges in nanoparticle characterization, which are currently unaddressed by other size-based separation approaches. Compared to SEC, FFF separation is usually gentler and more protective for fragile particles in terms of degradation or aggregation. However, analyte-membrane interaction has been noted, which leads to sample loss and low recovery, especially when using conventional AF4 with focusing prior to separation [146]. The mitigation strategies have been described in literature to reduce the analyte-membrane interaction by careful selection of the liquid carrier, membrane type, and molecular weight cutoff (MWCO) [147,148]. The advancement in membrane manufacturing, including robustness, solvent compatibility, and surface properties, could smooth the AF4 methodology development as well. Additionally, using a non-focusing channel in AF4 (frit-inlet or dispersion channel) could potentially circumvent a membrane interaction issue for some vulnerable nanoparticles during the focusing step.

Numerous applications of FFF in the separation of nanoparticles from a complex matrix have demonstrated that FFF is a very promising purification technique where the particle integrity could be maintained during the separation process. However, the loading capacity is still a drawback for purification when a large volume is needed to yield sufficient material. This necessitates the development of instrumentation, such as preparative channels, and this combination with fraction collectors will make FFF applicable as a preparative system. Another challenge arises from the sample dilution due to the high flow rate in FFF. Efforts have been made to increase the sample concentration for the following analysis. Manufacturers have developed dilution control modules to extract up to 90% of the sample-free liquid carrier to waste and deliver the concentrated sample to the online detectors, thus improving the detector limit for the low-abundance analytes [131]. Alternatively, the enrichment step post-FFF separation can be employed to concentrate the FFF fractions offline. For example, filtration was reported to concentrate the FFF fractions prior to offline quantitative analysis by CE [70]. Online sample enrichment could be another future direction to improve the performance of FFF both quantitatively and qualitatively. A solid-phase extraction (SPE) pre-column was introduced into the online enrichment system, combined with atmospheric pressure chemical ionization-mass spectrometry (APCI-MS) [149]. The next challenge is to explore the online coupling of AF4 with complementary detection techniques, such as high-resolution MS, to obtain simultaneous structure and compositional information, or with orthogonal separation techniques, such as LC and CE, to achieve improved selectivity from multi-dimensional separation. These will benefit from the development of online enrichment systems and customized interfaces. Such efforts have been made to achieve automated online isolation and fractionation for nanosized biomacromolecules by online coupled immunoaffinity chromatography-AF4 [150], demonstrating the potential of FFF in multi-dimensional analysis. In combination with hyphenated techniques, the investigation scope of FFF could be remarkably improved towards nanoparticles with higher complexity.

Intelligent software advancement is also an essential part of the development of technology, which will make the technique more user-friendly and thus facilitate the data analysis and interpretation, especially for non-spherical particle analysis. At the same time, substantial efforts have been made to develop standard analytical methods or protocols to guide FFF development in the pharmaceutical industry and address the regulatory expectations [35,151]. Standardized FFF methods for each type of nanoparticle would be helpful to flatten the learning curve for new users and make the technique readily accessible for researchers. Therefore, challenges and opportunities co-exist in the field of FFF. The remarkable versatility of FFF makes it a highly promising analytical platform for comprehensive physicochemical characterization of nanomedicines in pre-clinical investigation, product development, and quantity control of manufacturing. Moving forward, continuous improvements in instrumentation and software are expected to create more opportunities for FFF in different fields, in addition to drug delivery.

## 4. Summary

Multi-detector AF4 (MD-AF4) represents a multi-attribute characterization platform, which has been demonstrated to be a promising, powerful, and versatile analytical technique for size-dependent characterization of drug delivery nanoparticles. In particular, MD-AF4 is employed to (i) measure particle size distribution of highly heterogeneous samples; (ii) evaluate the morphology through shape factor; (iii) determine size-dependent payloads or drug-nanoparticle interactions; and (iv) study drug release and stability of the nanoparticle in the formulation buffer or biological matrix. For nanoparticles used in drug delivery applications, their size, shape, protein binding, and release kinetics play a significant role in biodistribution, off-target toxicities, and ultimately safety and efficacy. Those critical quality attributes must be carefully monitored during formulation development and manufacturing control. As a gentle size-based separation technique, AF4 is positioned to be the foremost technique for such analysis where traditional SEC fails, i.e., separation and characterization of lipid NPs, liposomes, EVs, and gene vectors. As presented in this review, MD-AF4 can be used either as a single technique or in combination with other complementary analytical techniques for the physical-chemical characterization of drug delivery nanoparticles. The wide applications of AF4 and its unique separation nature make MD-AF4 an enabling technology platform to provide high resolution and size-dependent characterization for various nanoparticles. AF4 will be more widely used in the pharmaceutical industry with the advancement of instrumentation and software, as well as regulatory guidance.

## Figures and Tables

**Table 1:** Summary of FFF sub-techniques.

Sub-Techniques of FFF	External Field	Physicochemical Property
Thermal FFF (ThFFF)	Thermal gradient	Soret coefficient
Sedimentation FFF (SeFFF)	Gravity/Centrifugal force	Effective mass (density)
Electrical FFF (ElFFF)	Electric field	Electrophoretic mobility
Magnetic FFF (MgFFF)	Magnetic field	Magnetic properties
Flow FFF (FlFFF)	Cross flow	Diffusion coefficient

**Table 2:** Example of FFF applications in drug delivery nanoparticles.

Nanoparticles	FFF Technique and Applications	Key Results	Ref
Lipid-based nanoparticles	AF4 with offline NTA and LC-MS for doxorubicin liposome formulations	Particle size distribution of the liposome and drug-to-lipid ratios were analyzed and compared across different doxorubicin formulations.	[53]
	AF4-MALS-DLS for peptide-liposome interaction	Selectivity of the peptide, quantity of the bound peptide, and size distribution and morphology of liposomes were revealed for understanding of structure-activity relationship.	[54]
	AF4-MALS-Gamma ray detector for liposome loaded with high energy alpha emitter (^212^Bi)	Liposome particle size and stability of encapsulation in the serum were studied.	[55]
	AF4-MALS for drug transfer assay to quantify retention of lipophilic model compounds	Transfer kinetics of lipophilic model compounds from donor liposomes to acceptor liposomes were elucidated at different lipid mass ratios, and with different vesicle morphology and lamellarity.	[56]
	AF4-MALS-RI for stability evaluation of liposomes against the intestinal bile salts in oral delivery application	Different mechanisms of entrapped calcein leakages were revealed.	[57]
	AF4-MALS-DLS-dRI-UV for liposome-plasma protein interaction (from albumin HDL and LDL	Liposomes were separated from albumin, and HDL, LHL, and the size were determined. The effect of the biolayer composition on liposome stability was also observed.	[58]
	AF4 with frit-inlet channel coupled with MALS to analyze LNP for RNA delivery	Frit-inlet channel enabled size and physical stability of LNP-RNA with great reliability and recovery.	[43]
Polymer-based nanoparticles	AF4-DLS-UV-FLD for enrofloxacin in PLGA nanoparticles	Comprehensive analysis of nanoparticle concentration (via UV), drug concentration (via FLD), and particle size distribution (via DLS). Unentrapped drug was easily removed via crossflow.	[59]
	AF4-MALS for protein-conjugated polysaccharides	Complementary analysis by SEC-MALS and AF4-MALS revealed heterogeneity in conformation and aggregation of the conjugates from molar mass and size determination.	[60]
	AF4-RI-FLD-DLS for polymer micelles in vitro stability	AF4 enabled the separation of polymer micelles from plasma protein and can be used to study the in vitro instability of drug-loaded nanoparticles.	[61]
	AF4-MALS-DLS for PEG-PDLLA polymersomes	Insights in size and shape of polymersomes via combination of MALS and DLS, and whether they are empty or loaded.	[62]
	AF4-RI for PAMAM dendrimers	Separate impurities (i.e., missing arm) and aggregates from PAMAM main populations and monitor interactions of PAMAM dendrimers with BSA.	[63]
	AF4-MALS-RI-UV for PEI-Mal dendrimers	Characterization of crossflow pathway enabled quantification of free, unencapsulated dye in addition to molar mass distributions.	[64]
Viral vectors and Virus-like Nanoparticles	AF4-MALS-DLS-UV-FLD-RI for VLPs derived from human polyoma JC virus	Comprehensive analysis of VLP molar mass and radius (via MALS), hydrodynamic radius (via DLS), concentration (via RI), sample composition and concentration (via UV), and improved small molecule limit of detection (via FLD).	[49]
	AF4-MALS-UV & ElFFF-MALS-UV of bacteriophage-like VLPs	Complementary analysis by ElFFF and AF4 obtained size and electrophoretic mobility of three VLPs.	[65]
Extracellular vesicles	AF4-UV-MALS for characterization of EVs from urine and comparison with ultrafiltration combined with SEC method	AF4-UV-MALS was demonstrated to be a straightforward and reproducible method for determining size, amount, and purity of isolated urinary EVs.	[66]
	AF4-UV-MALS combined with batch DLS and NTA for size separation, characterization and quantification of exosomes	Fractionation quality of exosomes was significantly influenced by crossflow conditions and channel thickness where focusing time has less impact. AF4-UV-MALS and DLS both showed the presence of two particle subpopulations. Compared to DLS and AF4-MALS, NTA overestimated the size and number density for the larger exosome population.	[67]
	AF4-UV-DLS with EM imaging for identification of subsets of EVs	Two exosome subpopulations and one non-membrane NPs exomere were discovered and identified	[68]
	AF4 and nanoflow-LC-ESI-MS/MS for size dependent lipidomic analysis of urinary exosomes	AF4 enabled the fractionation of exosomes with different sizes that originated from different types of cells. Degree of lipid increase was more significant in the smaller fractions, indicating that AF4 is capable of screening of urinary exosomes in cancer patients.	[69]
	Offline coupling of AF4 and CE for separation of EVs	EVs could be resolved from free proteins and high-density lipoproteins by AF4 and further separated from the low-density lipoproteins co-eluted in AF4 by offline CE.	[70]
	Orthogonal approach of ultracentrifugation and HF5-MALS-UV-FLD for purification and mapping of EV subtypes	Size, abundance, and DNA/protein content of the large and small EVs were characterized by HF5-MALS-UV-FLD as the second dimension, showing potential in sorting particles with different sizes and contents.	[71]
	EAF4 hyphenated with MALS and NTA for fast and purification-free characterization of NPs	EAF4 provided online sample purification and simultaneous access to size and Zeta-potential; high resolution size and number concentration was achieved by hyphenation of EAF4 with MALS and NTA.	[72]
Inorganic nanoparticles	AF4-MALS-DLS-ICPMS for quantitative characterization of GNPs	Mixtures of three GNPs were separated by AF4 and then each fraction was quantified by ICPMS. Both geometric diameters and hydrodynamic diameters were determined online by MALS and DLS.	[73]
	AF4 for characterization of elution behavior of non-spherical GNPs	Elution behavior of the GNPs with three different morphologies was studied by AF4 and particle size was compared with DLS and TEM.	[74]
	AF4-MALS-UV-RI for characterization and stability evaluation of drug-loaded metal-organic framework (MOF) NPs	Empty and drug-loaded nanoMOFs were studied in terms of particle size distribution and stability. Detection of aggregate formation and monitoring of nanoMOF morphological changes indicates their interaction with the drug molecules.	[75]

## Data Availability

Not applicable.

## References

[1] Sharma K., Koirala A., Nicolopoulos K., Chiu C., Wood N., Britton P.N. (2021). Vaccines for COVID-19: Where do we stand in 2021?. Paediatr. Respir. Rev..

[2] Hussain A., Yang H., Zhang M., Liu Q., Alotaibi G., Irfan M., He H., Chang J., Liang X.J., Weng Y. (2022). Mrna vaccines for COVID-19 and diverse diseases. J. Control Release.

[3] Rauf A., Abu-Izneid T., Khalil A.A., Hafeez N., Olatunde A., Rahman M., Semwal P., Al-Awthan Y.S., Bahattab O.S., Khan I.N. (2022). Nanoparticles in clinical trials of COVID-19: An update. Int. J. Surg..

[4] Jahangirian H., Lemraski E.G., Webster T.J., Rafiee-Moghaddam R., Abdollahi Y. (2017). A review of drug delivery systems based on nanotechnology and green chemistry: Green nanomedicine. Int. J. Nanomed..

[5] Patra J.K., Das G., Fraceto L.F., Campos E.V.R., Rodriguez-Torres M.D.P., Acosta-Torres L.S., Diaz-Torres L.A., Grillo R., Swamy M.K., Sharma S. (2018). Nano based drug delivery systems: Recent developments and future prospects. J. Nanobiotechnol..

[6] Yao Y., Zhou Y., Liu L., Xu Y., Chen Q., Wang Y., Wu S., Deng Y., Zhang J., Shao A. (2020). Nanoparticle-based drug delivery in cancer therapy and its role in overcoming drug resistance. Front. Mol. Biosci..

[7] Dos Santos Rodrigues B., Lakkadwala S., Kanekiyo T., Singh J. (2019). Development and screening of brain-targeted lipid-based nanoparticles with enhanced cell penetration and gene delivery properties. Int. J. Nanomed..

[8] Ickenstein L.M., Garidel P. (2019). Lipid-based nanoparticle formulations for small molecules and rna drugs. Expert. Opin. Drug. Deliv..

[9] Thi T.T.H., Suys E.J.A., Lee J.S., Nguyen D.H., Park K.D., Truong N.P. (2021). Lipid-based nanoparticles in the clinic and clinical trials: From cancer nanomedicine to COVID-19 vaccines. Vaccines.

[10] Sur S., Rathore A., Dave V., Reddy K.R., Chouhan R.S., Sadhu V. (2019). Recent developments in functionalized polymer nanoparticles for efficient drug delivery system. Nano-Struct. Nano-Objects.

[11] Charelli L.E., de Mattos G.C., de Jesus Sousa-Batista A., Pinto J.C., Balbino T.A. (2022). Polymeric nanoparticles as therapeutic agents against coronavirus disease. J. Nanopart Res..

[12] Ghezzi M., Pescina S., Padula C., Santi P., Del Favero E., Cantu L., Nicoli S. (2021). Polymeric micelles in drug delivery: An insight of the techniques for their characterization and assessment in biorelevant conditions. J. Control Release.

[13] Jeevanandam J., Pal K., Danquah M.K. (2019). Virus-like nanoparticles as a novel delivery tool in gene therapy. Biochimie.

[14] Chung Y.H., Cai H., Steinmetz N.F. (2020). Viral nanoparticles for drug delivery, imaging, immunotherapy, and theranostic applications. Adv. Drug. Deliv. Rev..

[15] Walker S., Busatto S., Pham A., Tian M., Suh A., Carson K., Quintero A., Lafrence M., Malik H., Santana M.X. (2019). Extracellular vesicle-based drug delivery systems for cancer treatment. Theranostics.

[16] Herrmann I.K., Wood M.J.A., Fuhrmann G. (2021). Extracellular vesicles as a next-generation drug delivery platform. Nat. Nanotechnol..

[17] Ghosn Y., Kamareddine M.H., Tawk A., Elia C., El Mahmoud A., Terro K., El Harake N., El-Baba B., Makdessi J., Farhat S. (2019). Inorganic nanoparticles as drug delivery systems and their potential role in the treatment of chronic myelogenous leukaemia. Technol. Cancer Res. Treat..

[18] Shi Z., Zhou Y., Fan T., Lin Y., Zhang H., Mei L. (2020). Inorganic nano-carriers based smart drug delivery systems for tumor therapy. Smart Mater. Med..

[19] (2022). Drug Products, Including Biological Products, That Contain Nanomaterials—Guidance for Industry.

[20] (2018). Liposome Drug Products Chemistry, Manufacturing, and Controls; Human Pharmacokinetics and Bioavailability; and Labeling Documentation.

[21] (2013). Reflection Paper on the Data Requirements for Intravenous Liposomal Products Developed with Reference to an Innovator Liposomal Product.

[22] Giddings J.C. (1966). A new separation concept based on a coupling of concentration and flow nonuniformities. Sep. Sci..

[23] Plavchak C.L., Smith W.C., Bria C.R.M., Williams S.K.R. (2021). New advances and applications in field-flow fractionation. Annu. Rev. Anal. Chem..

[24] Zattoni A., Roda B., Borghi F., Marassi V., Reschiglian P. (2014). Flow field-flow fractionation for the analysis of nanoparticles used in drug delivery. J. Pharm. Biomed. Anal..

[25] Hovingh M.E., Thompson G.H., Giddings J.C. (1970). Column parameters in thermal field-flow fractionation. Anal. Chem..

[26] Liu G., Giddings J.C. (1992). Separation of particles in aqueous suspensions by thermal field-flow fractionation—Measurement of thermal-diffusion coefficients. Chromatographia.

[27] Giddings J.C., Yang F.J.F., Myers M.N. (1974). Sedimentation field-flow fractionation. Anal. Chem..

[28] Chianéa T., Assidjo N.E., Cardot P.J.P. (2000). Sedimentation field-flow-fractionation: Emergence of a new cell separation methodology. Talanta.

[29] Williams P.S., Carpino F., Zborowski M. (2009). Magnetic nanoparticle drug carriers and their study by quadrupole magnetic field-flow fractionation. Mol. Pharm..

[30] Caldwell K.D., Gao Y.S. (1993). Electrical field-flow fractionation in particle separation. 1. Monodisperse standards. Anal. Chem..

[31] Johann C., Elsenberg S., Schuch H., Rosch U. (2015). Instrument and method to determine the electrophoretic mobility of nanoparticles and proteins by combining electrical and flow field-flow fractionation. Anal. Chem..

[32] Contado C. (2017). Field flow fractionation techniques to explore the “nano-world”. Anal. Bioanal. Chem..

[33] Giddings J.C., Yang F.J., Myers M.N. (1976). Flow-field-flow fractionation: A versatile new separation method. Science.

[34] Wahlund K.G., Giddings J.C. (1987). Properties of an asymmetrical flow field-flow fractionation channel having one permeable wall. Anal. Chem..

[35] Caputo F., Mehn D., Clogston J.D., Rosslein M., Prina-Mello A., Borgos S.E., Gioria S., Calzolai L. (2021). Asymmetric-flow field-flow fractionation for measuring particle size, drug loading and (in)stability of nanopharmaceuticals. The joint view of european union nanomedicine characterization laboratory and national cancer institute—Nanotechnology characterization laboratory. J. Chromatogr. A.

[36] Wahlund K.G. (2013). Flow field-flow fractionation: Critical overview. J. Chromatogr. A.

[37] Moon M.H., Hwang I. (2007). Hydrodynamic vs. Focusing relaxation in asymmetrical flow field-flow fractionation. J. Liq. Chromatogr. Relat. Technol..

[38] Fuentes C., Choi J., Zielke C., Penarrieta J.M., Lee S., Nilsson L. (2019). Comparison between conventional and frit-inlet channels in separation of biopolymers by asymmetric flow field-flow fractionation. Analyst.

[39] Mildner R., Hak S., Parot J., Hyldbakk A., Borgos S.E., Some D., Johann C., Caputo F. (2021). Improved multidetector asymmetrical-flow field-flow fractionation method for particle sizing and concentration measurements of lipid-based nanocarriers for rna delivery. Eur. J. Pharm. Biopharm..

[40] Elvang P.A., Stein P.C., Bauer-Brandl A., Brandl M. (2017). Characterization of co-existing colloidal structures in fasted state simulated fluids fassif: A comparative study using af4/malls, dls and dosy. J. Pharm. Biomed. Anal..

[41] Champagne J. Vlp Characterization: The Light Scattering Biophysical Toolbox. https://www.wyatt.com/library/webinars/vlp-characterization-light-scattering-biophysical-toolbox.html.

[42] Gobalasingham N. Expanding the Characterization Toolkit with fff-mals: Developments, Techniques, and Applications. https://www.wyatt.com/library/webinars/expanding-the-characterization-toolkit-with-fff-mals-developments-techniques-and-applications.html?utm_source=lcgc&utm_medium=digital-ad&utm_campaign=resource-center-08-2021.

[43] Gioria S., Caputo F., Urban P., Maguire C.M., Bremer-Hoffmann S., Prina-Mello A., Calzolai L., Mehn D. (2018). Are existing standard methods suitable for the evaluation of nanomedicines: Some case studies. Nanomedicine.

[44] Hinna A., Steiniger F., Hupfeld S., Brandl M., Kuntsche J. (2014). Asymmetrical flow field-flow fractionation with on-line detection for drug transfer studies: A feasibility study. Anal. Bioanal. Chem..

[45] Citkowicz A., Petry H., Harkins R.N., Ast O., Cashion L., Goldmann C., Bringmann P., Plummer K., Larsen B.R. (2008). Characterization of virus-like particle assembly for DNA delivery using asymmetrical flow field-flow fractionation and light scattering. Anal. Biochem..

[46] Janwitayanuchit W., Suwanborirux K., Patarapanich C., Pummangura S., Lipipun V., Vilaivan T. (2003). Synthesis and anti-herpes simplex viral activity of monoglycosyl diglycerides. Phytochemistry.

[47] Nilsson L. (2013). Separation and characterization of food macromolecules using field-flow fractionation: A review. Food Hydrocoll..

[48] Fraunhofer W., Winter G. (2004). The use of asymmetrical flow field-flow fractionation in pharmaceutics and biopharmaceutics. Eur. J. Pharm. Biopharm..

[49] Wagner M., Holzschuh S., Traeger A., Fahr A., Schubert U.S. (2014). Asymmetric flow field-flow fractionation in the field of nanomedicine. Anal. Chem..

[50] Wyatt P.J. (2014). Measurement of special nanoparticle structures by light scattering. Anal. Chem..

[51] Mogensen K.B., Kneipp K. (2014). Size-dependent shifts of plasmon resonance in silver nanoparticle films using controlled dissolution: Monitoring the onset of surface screening effects. J. Phys. Chem. C.

[52] Thomsen T., Ayoub A.B., Psaltis D., Klok H.A. (2021). Fluorescence-based and fluorescent label-free characterization of polymer nanoparticle decorated t cells. Biomacromolecules.

[53] Ansar S.M., Mudalige T. (2019). Characterization of doxorubicin liposomal formulations for size-based distribution of drug and excipients using asymmetric-flow field-flow fractionation (af4) and liquid chromatography-mass spectrometry (lc-ms). Int. J. Pharm..

[54] Iavicoli P., Urban P., Bella A., Ryadnov M.G., Rossi F., Calzolai L. (2015). Application of asymmetric flow field-flow fractionation hyphenations for liposome-antimicrobial peptide interaction. J. Chromatogr. A.

[55] Huclier-Markai S., Grivaud-Le Du A., N’Tsiba E., Montavon G., Mougin-Degraef M., Barbet J. (2018). Coupling a gamma-ray detector with asymmetrical flow field flow fractionation (af4): Application to a drug-delivery system for alpha-therapy. J. Chromatogr. A.

[56] Hinna A.H., Hupfeld S., Kuntsche J., Bauer-Brandl A., Brandl M. (2016). Mechanism and kinetics of the loss of poorly soluble drugs from liposomal carriers studied by a novel flow field-flow fractionation-based drug release-/transfer-assay. J. Control Release.

[57] Bohsen M.S., Tychsen S.T., Kadhim A.A.H., Grohganz H., Treusch A.H., Brandl M. (2023). Interaction of liposomes with bile salts investigated by asymmetric flow field-flow fractionation (af4): A novel approach for stability assessment of oral drug carriers. Eur. J. Pharm. Sci..

[58] Holzschuh S., Kaess K., Fahr A., Decker C. (2016). Quantitative in vitro assessment of liposome stability and drug transfer employing asymmetrical flow field-flow fractionation (af4). Pharm. Res..

[59] Shakiba S., Astete C.E., Cueto R., Rodrigues D.F., Sabliov C.M., Louie S.M. (2021). Asymmetric flow field-flow fractionation (af4) with fluorescence and multi-detector analysis for direct, real-time, size-resolved measurements of drug release from polymeric nanoparticles. J. Control Release.

[60] Deng J.Z., Lin J., Chen M., Lancaster C., Zhuang P. (2022). Characterization of high molecular weight pneumococcal conjugate by sec-mals and af4-mals. Polymers.

[61] Liu Y., Fens M., Capomaccio R.B., Mehn D., Scrivano L., Kok R.J., Oliveira S., Hennink W.E., van Nostrum C.F. (2020). Correlation between in vitro stability and pharmacokinetics of poly(epsilon-caprolactone)-based micelles loaded with a photosensitizer. J. Control Release.

[62] Wauters A.C., Pijpers I.A.B., Mason A.F., Williams D.S., Tel J., Abdelmohsen L., van Hest J.C.M. (2019). Development of morphologically discrete peg-pdlla nanotubes for precision nanomedicine. Biomacromolecules.

[63] Lee S., Kwen H.D., Lee S.K., Nehete S.V. (2010). Study on elution behavior of poly(amidoamine) dendrimers and their interaction with bovine serum albumin in asymmetrical flow field-flow fractionation. Anal. Bioanal. Chem..

[64] Boye S., Polikarpov N., Appelhans D., Lederer A. (2010). An alternative route to dye-polymer complexation study using asymmetrical flow field-flow fractionation. J. Chromatogr. A.

[65] Shiri F., Petersen K.E., Romanov V., Zou Q., Gale B.K. (2020). Characterization and differential retention of q beta bacteriophage virus-like particles using cyclical electrical field-flow fractionation and asymmetrical flow field-flow fractionation. Anal. Bioanal. Chem..

[66] Oeyen E., Van Mol K., Baggerman G., Willems H., Boonen K., Rolfo C., Pauwels P., Jacobs A., Schildermans K., Cho W.C. (2018). Ultrafiltration and size exclusion chromatography combined with asymmetrical-flow field-flow fractionation for the isolation and characterisation of extracellular vesicles from urine. J. Extracell. Vesicles.

[67] Sitar S., Kejžar A., Pahovnik D., Kogej K., Tušek-Žnidarič M., Lenassi M., Žagar E. (2015). Size characterization and quantification of exosomes by asymmetrical-flow field-flow fractionation. Anal. Chem..

[68] Zhang H., Freitas D., Kim H.S., Fabijanic K., Li Z., Chen H., Mark M.T., Molina H., Martin A.B., Bojmar L. (2018). Identification of distinct nanoparticles and subsets of extracellular vesicles by asymmetric flow field-flow fractionation. Nat. Cell Biol..

[69] Yang J.S., Lee J.C., Byeon S.K., Rha K.H., Moon M.H. (2017). Size dependent lipidomic analysis of urinary exosomes from patients with prostate cancer by flow field-flow fractionation and nanoflow liquid chromatography-tandem mass spectrometry. Anal. Chem..

[70] Gao Z., Hutchins Z., Li Z., Zhong W. (2022). Offline coupling of asymmetrical flow field-flow fractionation and capillary electrophoresis for separation of extracellular vesicles. Anal. Chem..

[71] Marassi V., Maggio S., Battistelli M., Stocchi V., Zattoni A., Reschiglian P., Guescini M., Roda B. (2021). An ultracentrifugation—Hollow-fiber flow field-flow fractionation orthogonal approach for the purification and mapping of extracellular vesicle subtypes. J. Chromatogr. A.

[72] Drexel R., Siupa A., Carnell-Morris P., Carboni M., Sullivan J., Meier F. (2020). Fast and purification-free characterization of bio-nanoparticles in biological media by electrical asymmetrical flow field-flow fractionation hyphenated with multi-angle light scattering and nanoparticle tracking analysis detection. Molecules.

[73] Schmidt B., Loeschner K., Hadrup N., Mortensen A., Sloth J.J., Koch C.B., Larsen E.H. (2011). Quantitative characterization of gold nanoparticles by field-flow fractionation coupled online with light scattering detection and inductively coupled plasma mass spectrometry. Anal. Chem..

[74] Lee J., Goda E.S., Choi J., Park J., Lee S. (2020). Synthesis and characterization of elution behavior of nonspherical gold nanoparticles in asymmetrical flow field-flow fractionation (asflfff). J. Nanoparticle Res..

[75] Roda B., Marassi V., Zattoni A., Borghi F., Anand R., Agostoni V., Gref R., Reschiglian P., Monti S. (2018). Flow field-flow fractionation and multi-angle light scattering as a powerful tool for the characterization and stability evaluation of drug-loaded metal-organic framework nanoparticles. Anal. Bioanal. Chem..

[76] Hupfeld S., Moen H.H., Ausbacher D., Haas H., Brandl M. (2010). Liposome fractionation and size analysis by asymmetrical flow field-flow fractionation/multi-angle light scattering: Influence of ionic strength and osmotic pressure of the carrier liquid. Chem. Phys. Lipids.

[77] Akbarzadeh A., Rezaei-Sadabady R., Davaran S., Joo S.W., Zarghami N., Hanifehpour Y., Samiei M., Kouhi M., Nejati-Koshki K. (2013). Liposome: Classification, preparation, and applications. Nanoscale Res. Lett..

[78] Cevc G. (2012). Rational design of new product candidates: The next generation of highly deformable bilayer vesicles for noninvasive, targeted therapy. J. Control Release.

[79] Barenholz Y. (2012). Doxil(r)—The first fda-approved nano-drug: Lessons learned. J. Control Release.

[80] Chang H.I., Yeh M.K. (2012). Clinical development of liposome-based drugs: Formulation, characterization, and therapeutic efficacy. Int. J. Nanomed..

[81] Hupfeld S., Holsaeter A.M., Skar M., Frantzen C.B., Brandl M. (2006). Liposome size analysis by dynamic/static light scattering upon size exclusion-/field flow-fractionation. J. Nanosci. Nanotechnol..

[82] Yohannes G., Pystynen K.-H., Riekkola M.-L., Wiedmer S.K. (2006). Stability of phospholipid vesicles studied by asymmetrical flow field-flow fractionation and capillary electrophoresis. Anal. Chim. Acta.

[83] Kuntsche J., Decker C., Fahr A. (2012). Analysis of liposomes using asymmetrical flow field-flow fractionation: Separation conditions and drug/lipid recovery. J. Sep. Sci..

[84] Rades A.S.T., Mozafari M.R. (2006). Solid lipid nanoparticles. Nanocarrier Technologies: Frontiers of Nanotherapy.

[85] Jenning V., Thunemann A.F., Gohla S.H. (2000). Characterisation of a novel solid lipid nanoparticle carrier system based on binary mixtures of liquid and solid lipids. Int. J. Pharm..

[86] Kristen A.V., Ajroud-Driss S., Conceição I., Gorevic P., Kyriakides T., Obici L. (2018). Patisiran, an rnai therapeutic for the treatment of hereditary transthyretin-mediated amyloidosis. Neurodegener. Dis. Manag..

[87] Baden L.R., El Sahly H.M., Essink B., Kotloff K., Frey S., Novak R., Diemert D., Spector S.A., Rouphael N., Creech C.B. (2021). Efficacy and safety of the mrna-1273 SARS-CoV-2 vaccine. N. Engl. J. Med..

[88] Polack F.P., Thomas S.J., Kitchin N., Absalon J., Gurtman A., Lockhart S., Perez J.L., Perez Marc G., Moreira E.D., Zerbini C. (2020). Safety and efficacy of the bnt162b2 mrna COVID-19 vaccine. N. Engl. J. Med..

[89] Pardi N., Hogan M.J., Porter F.W., Weissman D. (2018). Mrna vaccines—A new era in vaccinology. Nat. Rev. Drug. Discov..

[90] Parot J., Caputo F., Mehn D., Hackley V.A., Calzolai L. (2020). Physical characterization of liposomal drug formulations using multi-detector asymmetrical-flow field flow fractionation. J. Control Release.

[91] Chen S., Tam Y.Y.C., Lin P.J.C., Sung M.M.H., Tam Y.K., Cullis P.R. (2016). Influence of particle size on the in vivo potency of lipid nanoparticle formulations of sirna. J. Control Release.

[92] Jia X., Liu Y., Wagner A.M., Chen M., Zhao Y., Smith K.J., Some D., Abend A.M., Pennington J. (2021). Enabling online determination of the size-dependent rna content of lipid nanoparticle-based rna formulations. J. Chromatogr. B Anal. Technol. Biomed. Life Sci..

[93] Quattrini F., Berrecoso G., Crecente-Campo J., Alonso M.J. (2021). Asymmetric flow field-flow fractionation as a multifunctional technique for the characterization of polymeric nanocarriers. Drug. Deliv. Transl. Res..

[94] Zhao H., Lin Z.Y., Yildirimer L., Dhinakar A., Zhao X., Wu J. (2016). Polymer-based nanoparticles for protein delivery: Design, strategies and applications. J. Mater. Chem. B.

[95] Shakiba S., Shariati S., Wu H., Astete C.E., Cueto R., Fini E.H., Rodrigues D.F., Sabliov C.M., Louie S.M. (2022). Distinguishing nanoparticle drug release mechanisms by asymmetric flow field–flow fractionation. J. Control Release.

[96] Sadat Tabatabaei Mirakabad F., Nejati-Koshki K., Akbarzadeh A., Yamchi M.R., Milani M., Zarghami N., Zeighamian V., Rahimzadeh A., Alimohammadi S., Hanifehpour Y. (2014). Plga-based nanoparticles as cancer drug delivery systems. Asian Pac. J. Cancer Prev..

[97] Alvi M., Yaqoob A., Rehman K., Shoaib S.M., Akash M.S.H. (2022). Plga-based nanoparticles for the treatment of cancer: Current strategies and perspectives. AAPS Open.

[98] Yadav N., Francis A.P., Priya V.V., Patil S., Mustaq S., Khan S.S., Alzahrani K.J., Banjer H.J., Mohan S.K., Mony U. (2022). Polysaccharide-drug conjugates: A tool for enhanced cancer therapy. Polymers.

[99] Dacoba T.G., Omange R.W., Li H., Crecente-Campo J., Luo M., Alonso M.J. (2019). Polysaccharide nanoparticles can efficiently modulate the immune response against an hiv peptide antigen. ACS Nano.

[100] Klein M., Menta M., Dacoba T.G., Crecente-Campo J., Alonso M.J., Dupin D., Loinaz I., Grassl B., Séby F. (2020). Advanced nanomedicine characterization by dls and af4-uv-mals: Application to a hiv nanovaccine. J. Pharm. Biomed. Anal..

[101] Biemans R., Micoli F., Romano M.R. (2020). Glycoconjugate vaccines, production and characterization. Recent Trends in Carbohydrate Chemistry.

[102] Barth H.G., Carlin F.J. (1984). A review of polymer shear degradation in size-exclusion chromatography. J. Liq. Chromatogr..

[103] Kuntsche J., Horst J.C., Bunjes H. (2011). Cryogenic transmission electron microscopy (cryo-tem) for studying the morphology of colloidal drug delivery systems. Int. J. Pharm..

[104] Truong N.P., Whittaker M.R., Mak C.W., Davis T.P. (2015). The importance of nanoparticle shape in cancer drug delivery. Expert. Opin. Drug. Deliv..

[105] Wong C.K., Stenzel M.H., Thordarson P. (2019). Non-spherical polymersomes: Formation and characterization. Chem. Soc. Rev..

[106] Burchard W., Schmidt M., Stockmayer W.H. (1980). Information on polydispersity and branching from combined quasi-elastic and intergrated scattering. Macromolecules.

[107] Kok C.M., Rudin A. (1981). Relationship between the hydrodynamic radius and the radius of gyration of a polymer in solution. Die Makromol. Chem. Rapid Commun..

[108] Tomalia D.A., Naylor A.M., Goddard W.A. (1990). Starburst dendrimers: Molecular-level control of size, shape, surface chemistry, topology, and flexibility from atoms to macroscopic matter. Angew. Chem. Int. Ed. Engl..

[109] Wang J., Li B., Qiu L., Qiao X., Yang H. (2022). Dendrimer-based drug delivery systems: History, challenges, and latest developments. J. Biol. Eng..

[110] Kootstra N.A., Verma I.M. (2003). Gene therapy with viral vectors. Annu. Rev. Pharmacol. Toxicol..

[111] Shah P.B., Losordo D.W. (2005). Non-viral vectors for gene therapy: Clinical trials in cardiovascular disease. Advances in Genetics.

[112] Eisenman D. (2019). The united states’ regulatory environment is evolving to accommodate a coming boom in gene therapy research. Appl. Biosaf..

[113] Lee C.S., Bishop E.S., Zhang R., Yu X., Farina E.M., Yan S., Zhao C., Zheng Z., Shu Y., Wu X. (2017). Adenovirus-mediated gene delivery: Potential applications for gene and cell-based therapies in the new era of personalized medicine. Genes. Dis..

[114] Rodriguez D.A., Vader P. (2022). Extracellular vesicle-based hybrid systems for advanced drug delivery. Pharmaceutics.

[115] Jayasinghe M.K., Tan M., Peng B., Yang Y., Sethi G., Pirisinu M., Le M.T.N. (2021). New approaches in extracellular vesicle engineering for improving the efficacy of anti-cancer therapies. Semin. Cancer Biol..

[116] Ferreira D., Moreira J.N., Rodrigues L.R. (2022). New advances in exosome-based targeted drug delivery systems. Crit. Rev. Oncol. Hematol..

[117] Amiri A., Bagherifar R., Ansari Dezfouli E., Kiaie S.H., Jafari R., Ramezani R. (2022). Exosomes as bio-inspired nanocarriers for rna delivery: Preparation and applications. J. Transl. Med..

[118] De Jong O.G., Kooijmans S.A.A., Murphy D.E., Jiang L., Evers M.J.W., Sluijter J.P.G., Vader P., Schiffelers R.M. (2019). Drug delivery with extracellular vesicles: From imagination to innovation. Acc. Chem. Res..

[119] Zhang Q., Zhang H., Ning T., Liu D., Deng T., Liu R., Bai M., Zhu K., Li J., Fan Q. (2020). Exosome-delivered c-met sirna could reverse chemoresistance to cisplatin in gastric cancer. Int. J. Nanomed..

[120] Pascucci L., Cocce V., Bonomi A., Ami D., Ceccarelli P., Ciusani E., Vigano L., Locatelli A., Sisto F., Doglia S.M. (2014). Paclitaxel is incorporated by mesenchymal stromal cells and released in exosomes that inhibit in vitro tumor growth: A new approach for drug delivery. J. Control Release.

[121] Thery C., Witwer K.W., Aikawa E., Alcaraz M.J., Anderson J.D., Andriantsitohaina R., Antoniou A., Arab T., Archer F., Atkin-Smith G.K. (2018). Minimal information for studies of extracellular vesicles 2018 (misev2018): A position statement of the international society for extracellular vesicles and update of the misev2014 guidelines. J. Extracell. Vesicles.

[122] Witwer K.W., Goberdhan D.C., O’Driscoll L., Thery C., Welsh J.A., Blenkiron C., Buzas E.I., Di Vizio D., Erdbrugger U., Falcon-Perez J.M. (2021). Updating misev: Evolving the minimal requirements for studies of extracellular vesicles. J. Extracell. Vesicles.

[123] Yang J.S., Kim J.Y., Lee J.C., Moon M.H. (2019). Investigation of lipidomic perturbations in oxidatively stressed subcellular organelles and exosomes by asymmetrical flow field-flow fractionation and nanoflow ultrahigh performance liquid chromatography-tandem mass spectrometry. Anal. Chim. Acta.

[124] Wu B., Chen X., Wang J., Qing X., Wang Z., Ding X., Xie Z., Niu L., Guo X., Cai T. (2020). Separation and characterization of extracellular vesicles from human plasma by asymmetrical flow field-flow fractionation. Anal. Chim. Acta.

[125] Kim Y.B., Yang J.S., Lee G.B., Moon M.H. (2020). Evaluation of exosome separation from human serum by frit-inlet asymmetrical flow field-flow fractionation and multiangle light scattering. Anal. Chim. Acta.

[126] Li P., Kaslan M., Lee S.H., Yao J., Gao Z. (2017). Progress in exosome isolation techniques. Theranostics.

[127] Chia B.S., Low Y.P., Wang Q., Li P., Gao Z. (2017). Advances in exosome quantification techniques. TrAC Trends Anal. Chem..

[128] Anderson W., Kozak D., Coleman V.A., Jamting A.K., Trau M. (2013). A comparative study of submicron particle sizing platforms: Accuracy, precision and resolution analysis of polydisperse particle size distributions. J. Colloid. Interface Sci..

[129] Zhang H., Zhang H., Lyden D. (2018). A protocol for asymmetric-flow field-flow fractionation (af4) of small extracellular vesicles. Protocol Exchange.

[130] Kim Y.B., Lee G.B., Moon M.H. (2022). Size separation of exosomes and microvesicles using flow field-flow fractionation/multiangle light scattering and lipidomic comparison. Anal. Chem..

[131] Podzimek S., Johann C. (2021). Asymmetric flow field-flow fractionation: Current status, possibilities, analytical limitations and future trends. Chromatographia.

[132] Jia Y.-P., Ma B.-Y., Wei X.-W., Qian Z.-Y. (2017). The in vitro and in vivo toxicity of gold nanoparticles. Chin. Chem. Lett..

[133] Xu Z.P., Zeng Q.H., Lu G.Q., Yu A.B. (2006). Inorganic nanoparticles as carriers for efficient cellular delivery. Chem. Eng. Sci..

[134] Biener J., Wittstock A., Baumann T., Weissmüller J., Bäumer M., Hamza A. (2009). Surface chemistry in nanoscale materials. Materials.

[135] Kong F.Y., Zhang J.W., Li R.F., Wang Z.X., Wang W.J., Wang W. (2017). Unique roles of gold nanoparticles in drug delivery, targeting and imaging applications. Molecules.

[136] Wang W., Wang J., Ding Y. (2020). Gold nanoparticle-conjugated nanomedicine: Design, construction, and structure-efficacy relationship studies. J. Mater. Chem. B.

[137] Song M., Wang X., Li J., Zhang R., Chen B., Fu D. (2008). Effect of surface chemistry modification of functional gold nanoparticles on the drug accumulation of cancer cells. J. Biomed. Mater. Res. A.

[138] Zeng X., Liu G., Tao W., Ma Y., Zhang X., He F., Pan J., Mei L., Pan G. (2017). A drug-self-gated mesoporous antitumor nanoplatform based on ph-sensitive dynamic covalent bond. Adv. Funct. Mater..

[139] Farjadian F., Roointan A., Mohammadi-Samani S., Hosseini M. (2019). Mesoporous silica nanoparticles: Synthesis, pharmaceutical applications, biodistribution, and biosafety assessment. Chem. Eng. J..

[140] Anselmo A.C., Mitragotri S. (2015). A review of clinical translation of inorganic nanoparticles. AAPS J..

[141] Kong L., Qiu J., Sun W., Yang J., Shen M., Wang L., Shi X. (2017). Multifunctional pei-entrapped gold nanoparticles enable efficient delivery of therapeutic sirna into glioblastoma cells. Biomater. Sci..

[142] Wang F., Wang Y.C., Dou S., Xiong M.H., Sun T.M., Wang J. (2011). Doxorubicin-tethered responsive gold nanoparticles facilitate intracellular drug delivery for overcoming multidrug resistance in cancer cells. ACS Nano.

[143] Wang J., Giordani S., Marassi V., Roda B., Reschiglian P., Zattoni A. (2022). Quality control and purification of ready-to-use conjugated gold nanoparticles to ensure effectiveness in biosensing. Front. Sens..

[144] Marassi V., Zanoni I., Ortelli S., Giordani S., Reschiglian P., Roda B., Zattoni A., Ravagli C., Cappiello L., Baldi G. (2022). Native study of the behaviour of magnetite nanoparticles for hyperthermia treatment during the initial moments of intravenous administration. Pharmaceutics.

[145] Agostoni V., Anand R., Monti S., Hall S., Maurin G., Horcajada P., Serre C., Bouchemal K., Gref R. (2013). Impact of phosphorylation on the encapsulation of nucleoside analogues within porous iron(iii) metal-organic framework mil-100(fe) nanoparticles. J. Mater. Chem. B.

[146] Kowalkowski T., Sugajski M., Buszewski B. (2018). Impact of ionic strength of carrier liquid on recovery in flow field-flow fractionation. Chromatographia.

[147] Mudalige T.K., Qu H., Sanchez-Pomales G., Sisco P.N., Linder S.W. (2015). Simple functionalization strategies for enhancing nanoparticle separation and recovery with asymmetric flow field flow fractionation. Anal. Chem..

[148] Gigault J., Pettibone J.M., Schmitt C., Hackley V.A. (2014). Rational strategy for characterization of nanoscale particles by asymmetric-flow field flow fractionation: A tutorial. Anal. Chim. Acta.

[149] Valto P., Knuutinen J., Alen R. (2009). Evaluation of resin and fatty acid concentration levels by online sample enrichment followed by atmospheric pressure chemical ionization-mass spectrometry (APCI-MS). Environ. Sci. Pollut. Res. Int..

[150] Multia E., Liangsupree T., Jussila M., Ruiz-Jimenez J., Kemell M., Riekkola M.L. (2020). Automated on-line isolation and fractionation system for nanosized biomacromolecules from human plasma. Anal. Chem..

[151] Caputo F., Clogston J., Calzolai L., Rosslein M., Prina-Mello A. (2019). Measuring particle size distribution of nanoparticle enabled medicinal products, the joint view of euncl and nci-ncl. A step by step approach combining orthogonal measurements with increasing complexity. J. Control Release.

